# Computational prediction of the localization of microRNAs within their pre-miRNA

**DOI:** 10.1093/nar/gkt466

**Published:** 2013-06-08

**Authors:** Mickael Leclercq, Abdoulaye Banire Diallo, Mathieu Blanchette

**Affiliations:** ^1^School of Computer Science and McGill Centre for Bioinformatics, McGill University, Montreal, Quebec, Canada H3A2B2 and ^2^Laboratoire de bioinformatique du département informatique, Université du Québec À Montréal, Montreal, Quebec, Canada H2X3Y7

## Abstract

MicroRNAs (miRNAs) are short RNA species derived from hairpin-forming miRNA precursors (pre-miRNA) and acting as key posttranscriptional regulators. Most computational tools labeled as miRNA predictors are in fact pre-miRNA predictors and provide no information about the putative miRNA location within the pre-miRNA. Sequence and structural features that determine the location of the miRNA, and the extent to which these properties vary from species to species, are poorly understood. We have developed miRdup, a computational predictor for the identification of the most likely miRNA location within a given pre-miRNA or the validation of a candidate miRNA. MiRdup is based on a random forest classifier trained with experimentally validated miRNAs from miRbase, with features that characterize the miRNA–miRNA* duplex. Because we observed that miRNAs have sequence and structural properties that differ between species, mostly in terms of duplex stability, we trained various clade-specific miRdup models and obtained increased accuracy. MiRdup self-trains on the most recent version of miRbase and is easy to use. Combined with existing pre-miRNA predictors, it will be valuable for both *de novo* mapping of miRNAs and filtering of large sets of candidate miRNAs obtained from transcriptome sequencing projects. MiRdup is open source under the GPLv3 and available at http://www.cs.mcgill.ca/∼blanchem/mirdup/.

## INTRODUCTION

MicroRNAs (miRNAs) are short (generally 19–24 nucleotides) noncoding single-stranded RNA molecules that are involved in posttranscriptional regulation by targeting messenger RNAs (1–3). In animals, miRNAs expression is a multistep process (4): (i) transcription of the primary miRNAs (pri-miRNAs) by RNA polymerase II, (ii) cleavage of the pri-miRNA by Drosha and the RNAse III enzyme to isolate long hairpins called miRNA precursors (pre-miRNAs) and (iii) extraction by Dicer of the miRNA–miRNA* duplex from the pre-miRNA. In plants, Drosha and Dicer are replaced by Dicer Like 1 (5). The miRNA* is the complementary region of the miRNA on the other arm of the hairpin with a shift of 2 nt in the 5′ direction (6). After separation of the two strands of the duplex, the miRNA is mature and ready to be attached to the RISC complement. It then targets mRNAs by perfect or imperfect complementarity (7). In some cases, both miRNA and miRNA* are functional (8).

Over the past years, a number of studies have shown the involvement of miRNAs in most biological process (9). They are involved in developmental and physiological roles in animals and plants (10,11), such as differentiation of embryonic (12), muscle (13), skeletal (14), hematopoietic (15) and many other types of cells. They are also known to control cell death (16) and proliferation (17), insulin secretion (18) or lipid metabolism (19). Loss (20) and misregulation (21) of miRNAs also play an important role in several diseases (22,23), such as cancers (24,25). Finally, several studies revealed that organisms under various stress have a responsive miRNAs signature pattern, allowing resistance and adaptation (26–31). MiRNAs are even used by virus to infect hosts (32–34).

Although experimental techniques for unambiguous identification of miRNAs exist (35), they remain slow and expensive. Sequencing of short RNAs followed by mapping to a reference genome has become an approach of choice (36–38), but many small RNA molecules are unlikely to be miRNAs, while many true miRNAs are likely to be expressed only under rare circumstances not easily covered experimentally. For those reasons, computational prediction of miRNAs continues to play an important role in genomics.

Most miRNA prediction approaches rely, at least in part, on the specific hairpin shape of the secondary structure of the pre-miRNA (39). These include ProMir (40,41), TripletSVM (42), miRabela (43), miPred (44), SSCprofiler (45), microPred (46), HHMMiR (47), SplamiR (48), miRFinder (49), MiRenSVM, the only tools that handle multiloop hairpins (50), and many others. All these tools are trained on known miRNAs stored in MiRbase (51), a repository of miRNAs (mostly) experimentally validated. The prediction of the hairpin can be combined with comparative genomics approaches that posit that, in addition to their typical secondary structure, pre-miRNAs exhibit high sequence and structure conservation across species (52,53). However, most computational approaches labeled as miRNA predictors are actually pre-miRNA predictors, in the sense that they identify candidate genomic regions that may form pre-miRNAs but rarely attempt to determine the position of the miRNA itself within them.

Computationally predicted pre-miRNAs are often combined with high-throughput short-RNA sequencing data, in an attempt to determine which of the large number of expressed small RNAs may indeed be miRNAs. This kind of approach is challenging, though, as short reads may be incorrectly mapped, or may come from degradation products from the pre-miRNA, especially from the miRNA*, or from other types of RNA molecules. Predictions from deep sequencing can be obtained by considering the abundance and distribution of reads mapped to a candidate pre-miRNA, where read stacks and Dicer products mapped on a reference inform about the location of the miRNA. This strategy is used by miRdeep (6,54), miRdeep* (55), MIReNA (56) and miRanalyzer (57,58). However, lowly expressed miRNA, often lineage-specific (59) or condition-specific (60) ones, will be difficult to detect because Dicer products and the miRNA* are completely degraded.

To the best of our knowledge, only six mature miRNA predictors have been proposed to date. MIRcheck (28) identifies 20-nt regions of a given plant pre-miRNA using a predetermined set of rules and constraints. MiRalign (61) finds miRNAs positions by aligning pre-miRNAs with miRbase, thereby preventing from finding new miRNAs. ProMir (40) identifies human pre-miRNAs and their mature miRNAs by combining sequence and structural features in a paired hidden Markov model. MatureBayes (62) identifies 22-nt regions that are likely mature miRNA candidates based on sequence and secondary structure information using a Naive Bayes classifier. MaturePred (63) locates fixed-length miRNAs in plants based on miRNA–miRNAs* features and a support vector machine predictor. Finally, MiRmat (64) seeks Drosha and Dicer processing sites in vertebrates using a random forest predictor.

Although the recent research activity related to miRNA prediction shows the importance of the problem, existing tools have severe limitations. First, most tools are trained specifically on data from certain phyla [e.g. plants (28), humans (42,44) or viruses (33)], which limits their applicability. Second, most mature miRNA prediction tools seek mature miRNA of a fixed length, although in most species miRNAs lengths vary from 19 to 24 nt. Third, tools are typically trained once, at the time of publication, based on the training data available at that time. This means that they do not benefit from the rapid increase in the quality and quantity of experimentally verified miRNAs available. Finally, accessibility remains an issue, with ProMir 2 being unavailable and MaturePred, MiRalign and MiRmat being only available as web servers, which limits that usability for large-scale analyses.

In this article, we introduce miRdup (miRNA duplex), a tool for the validation of a candidate mature miRNA or the prediction of the precise position and length of the mature miRNA within a candidate pre-miRNA, based on a combination of sequence and structural features. We trained models separately on data from five lineages (mammals, fishes, arthropods, nematodes and plants), which increases species specificity and allows the discovery of features that distinguishes miRNAs from different species. The algorithm works on both single hairpin and multiloop pre-miRNAs. Finally, miRdup automatically downloads and trains on the latest miRbase release, to ensure it benefits from the most up-to-date data.

## MATERIALS AND METHODS

### Datasets

MiRNAs and pre-miRNAs sequences were downloaded from miRbase (http://www.mirbase.org/) (52) release 19, which contains 19 823 unique mature miRNAs/pre-miRNAs pairs. We note that until recently, miRNAs and miRNA* used to be annotated separately in mirBase and were thought to be functionally distinct, with the former playing a functional role and the latter being a non-functional by-product. This view has changed now owing to reports of functional activity of miRNAs* (65), and miRbase has stopped distinguishing between the miRNAs and miRNAs* (miRbase blog, 27 April 2011). We chose to follow this direction by considering all miRNAs and miRNAs* as functional, labeling them as either 3 prime or 5 prime depending on their location on the pre-miRNA hairpin. We note, however, that for >78% of cases, only one miRNA is annotated in a given pre-miRNA, with the complementary region not being annotated as functional.

For the purpose of training classifiers, negative sets of non-miRNAs were generated as follows. For each positive example (pair of miRNA and pre-miRNA), a negative example was generated by randomly relocating the miRNA along the same pre-miRNA sequence, preserving the miRNA’s length, but excluding the exact position of the true miRNA or of any other known miRNAs. Note that because of the non-deterministic selection of the negative examples, training results vary slightly from run to run. The complete training data set consisted of 19 823 positive examples and an equal number of negative examples.

### Feature vectors and training

Each training example was represented as a set of 100 features listed in Supplementary Table S1. The minimum free energy (MFE) and the secondary structure of the pre-miRNAs and the miRNA–miRNA* candidate duplexes were obtained with RNAfold and RNAduplex, from Vienna package (66), using default parameters. To perform the ranking of attributes and classifier training and evaluation, we used Weka and its libraries (67). All classifiers were trained using 10-fold cross-validation. Attributes ranking was performed using information gain evaluator (*InfoGain evaluator*) (68) with the *Ranker* search method (69) in Weka with default parameters and 10-fold cross validation. Ranker ranks attributes by their individual evaluations in conjunction with other attribute evaluators like ReliefF (70), GainRatio (71) and Entropy (72).

MiRdup uses a random forest classifier [a combination of decision tree predictors trained on a random subset of features sampled independently (73)], combined with the Adaboost M1 method (74). Adaboost is a machine learning meta-algorithm that is used in combination of many other machine learning algorithms to improve their performance (75). The random forest was trained with an unlimited maximum depth of the trees and 50 generated trees (Weka options: -I 50 -K 0 -S 1). Adaboost used 10 iterations, reweighting and a weight threshold of 100 (Weka options: -P 100 -S 1 -I 10). The other classifiers considered were (i) a support-vector machine (SVM) classifier (76), working with libSVM library (77), using with radial kernel (Weka options: -S 1 -K 2 -D 3 -G 0.0 -R 0.0 -N 0.5 -M 40.0 -C 1.0 -E 0.0010 -P 0.1) and (ii) the C4.5 decision tree classifier (J48) (78), trained with Adaboost (Weka options: AdaBoostM1 -P 100 -S 1 -I 10 -W trees.J48 – -C 0.25 -M 2).

The efficiency of a given classifier was measured as a function of its number of true positive (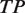), false positive (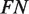), true negative (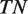) and false negative (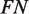) predictions. A classifier performance is typically measured by its sensitivity 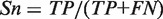 and specificity 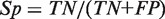, as well as by its total prediction accuracy 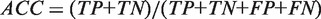 (44) and its Matthew’s correlation coefficient (79) 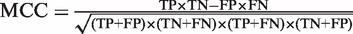 .

### MiRNA prediction

MiRdup can be used in two modes. In the validation mode, miRdup takes as input a pre-miRNA sequence and the position of a candidate miRNA, and returns a score that reflects the likelihood that the candidate is a true miRNA. In the prediction mode, the only input to miRdup is a pre-miRNA sequence, and it evaluates all possible miRNAs and reports the most likely miRNA-containing duplex. For each candidate, starting position *p* and length 16 ≤ *l* ≤ 30 on a pre-miRNA of length 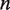, miRdup calculates score (*p, l*) using the random forest classifier, as described above. Although candidate miRNAs could simply be ranked based on these scores, we found that the following post-processing approach produced more accurate predictions. We first calculate, for each starting position *p*, the consensus scores for starting position score *S(p)* and ending position *E(p)*:

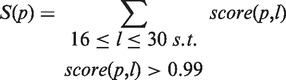


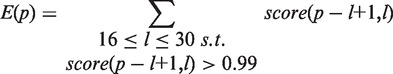

We then identify the position *p* and length *l* that results in the largest combined start and end position scores:

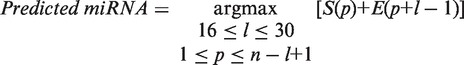



## RESULTS AND DISCUSSION

We developed miRdup, a classifier for the mature miRNA validation and identification in a given pre-miRNA sequence (see ‘Materials and Methods’ section). In the former case, miRdup assigns a score to a given candidate mature miRNAs within its pre-miRNA sequence. In the latter, it determines the most likely position of a mature miRNA within a given pre-miRNA sequence. MiRdup is based on a random forest binary classifier using a set of sequence and structural features of the candidate miRNA–miRNA* duplex. By training mirDup on lineage-specific subsets of miRbase, one obtains classifiers that can take advantage of miRNA features that are specific to that clade, which helps improve the accuracy of predictions. Here, we report on the accuracy of miRdup predictions in various settings, and contrast sequence and structure features that are informative for five selected clades: Mammals (mostly primates, rodents and carnivores), plants (mostly crucifers, maize and rice), fish (mostly zebrafish and fugu), arthropods (insects and crustaceans, etc.) and nematodes (*Caenorhabditis* and *Pristionchus pacificus*, etc.).

### Evaluation of individual predictive features

**Figure 1.**
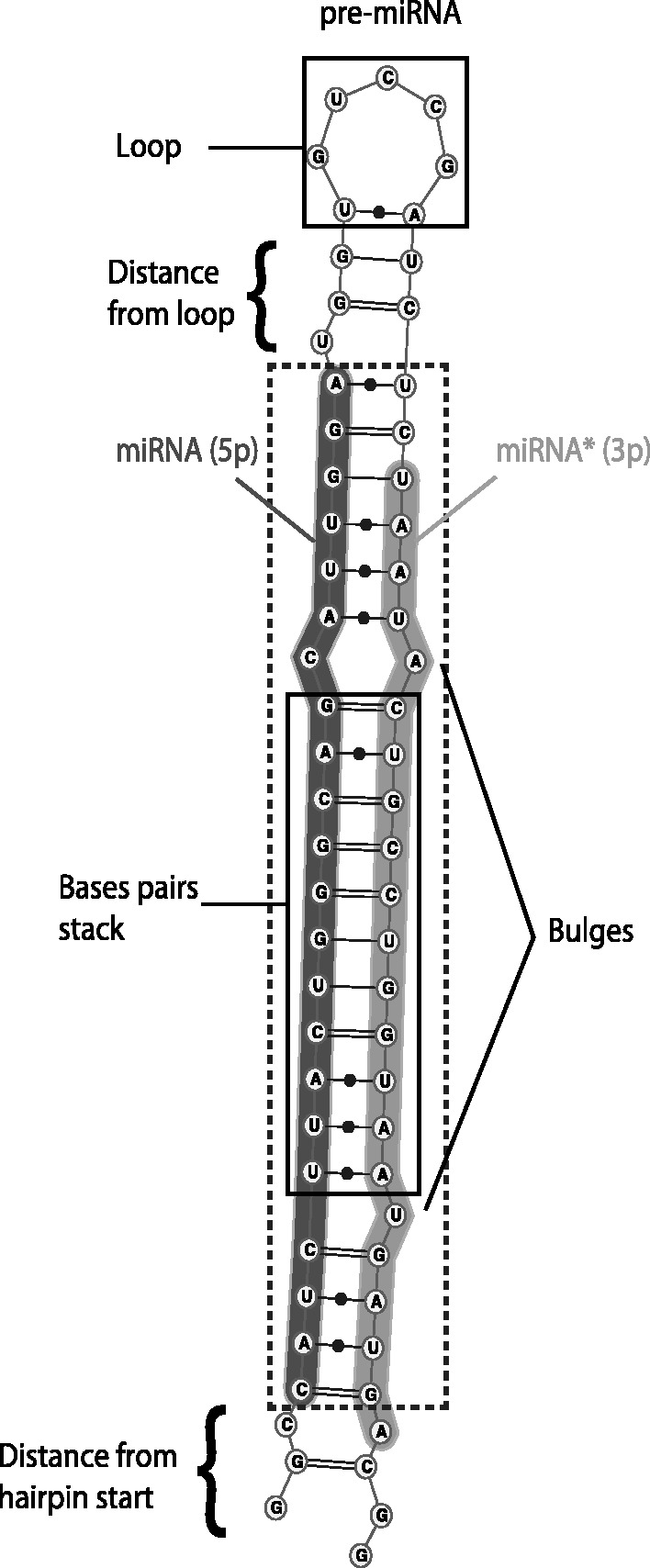
Structure of a pre-miRNA hairpin. The dashed box represents the duplex from which features are computed.

We evaluated a set of sequence and structural features (Supplementary Table S1), summarized in Table 1, which may potentially help characterize the position of the miRNA on the pre-miRNA hairpin. They were chosen based on previous studies focusing on miRNA prediction (42,44) and on the many properties that could characterize the duplex. These include numerical features describing the position and length of particular structural elements in the putative miRNA, such as bulges and bases pairs, or distance of the miRNA from the start/end of the hairpin (Figure 1). We also included summary statistics on the primary miRNA sequence (e.g. mononucleotide and dinucleotide frequencies) and the predicted secondary structure of the miRNA/miRNA* duplex [frequency of base pairs types (G-U, C-G or A-U), frequency of local sequence/structure triplets (A sequence/structure triplet corresponds to a nucleotide coupled by the sequence of presence/absence of base-pairing at that position and the two flanking positions. For example, ‘A(.(’ represents a case where a nucleotide A is in a bulge surrounded by two base pairs, and ‘U.(.’ means that a U is paired but its two neighbors are not.) (42) and MFE of the duplex]. We note that we also considered adding structural features based on ensembles of structures rather than MFE structures. However, these features did not prove more informative than their MFE-based counterparts and were not retained.

Features vary in their power to distinguish positive from negative examples. Identifying and removing uninformative features is often important to avoid overfitting and improve computational time (80), although this problem is less of an issue for algorithms based on decision trees and forests of random decision trees (81) than for SVMs (82). Features were ranked based on the information gain they provide (Table 2). We observe that the most influential features are those related to structural aspects of the miRNA–miRNA* duplex (number of base pairs, MFE, number/size of bulges, position of miRNA in the pre-miRNA hairpin loop). On the opposite, primary sequence features and triplet frequencies showed little discriminative power. We note that because our positive and negative examples were size-matched, miRNA length was not considered informative.

### Mature miRNAs exhibit species-specific properties

**Figure 2.**
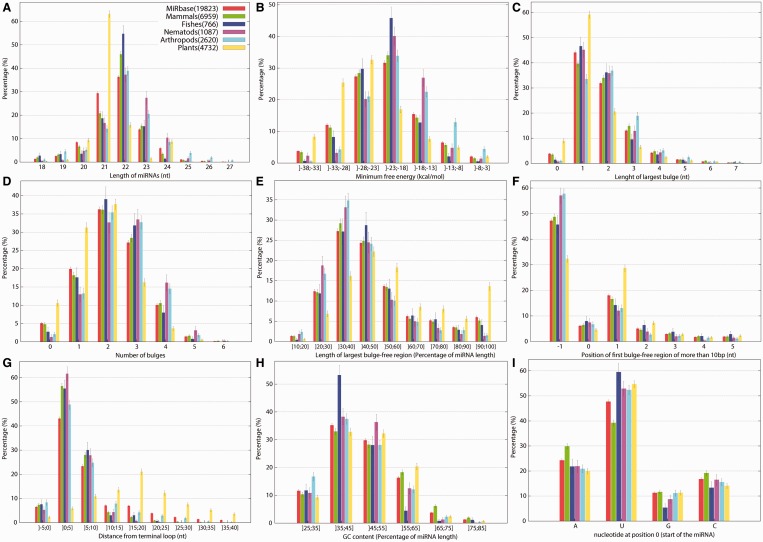
Properties of miRNAs from six different lineages: all eukaryotes (19 823 miRNAs), mammals (6959), fish (766), nematodes (1087), arthropods (2620) and plants (4732). Each panel shows the distribution of a selected feature. (**A**) MiRNA length (nt). (**B**) MFE of the miRNA–miRNA* duplex (kcal/mol). (**C**) Length of the largest bulge in the miRNA (nt). (**D**) Number of bulges in the miRNA–miRNA* duplex. (**E**) Length of longest bulge-free stem in the miRNA–miRNA* duplex. (**F**) Start position of the first 10 nt bulge-free stem in the miRNA–miRNA* duplex; −1 means no such region is present. (**G**) Distance to the terminal loop of the hairpin (nt). (**H**) miRNA GC-content. (**I**) Nucleotide type (A, U, G or C) at the first position of the miRNA.

We then assessed the power of each feature at distinguishing true miRNA from negative examples in specific lineages. Figure 2A–H shows distribution of feature values for some of those that vary significantly between lineages based on Kolmogorov–Smirnov test (*P*-value < 0.05 for at least one of the comparisons between lineage-specific distribution and the distribution obtained from all MirBase). The length of miRNAs varies significantly between species, where plant miRNA are generally 21 nt long and almost never 23 nt, while animal miRNAs have a broader, more regular miRNA length distribution with a mode at 22 nt (Figure 2A). Plant miRNAs also stand out with duplexes that are on average more stable (lower free energy) than animals’ (Figure 2B), while arthropods and, to a lesser extent, nematodes are often less stable. This is also reflected in various structural properties such as the presence of fewer and shorter bulges (Figure 2C, D). In fact, >13% of plant miRNAs have no bulge at all (100% base-paired positions, Figure 2E) and >33% have at least 10 consecutive base pairs starting at positions 0 (start) or 1 (Figure 2F), two properties that are much more rare in animals. Sixty percent to 90% of animal miRNAs are located within 10 bp of the terminal loop of the pre-miRNA (Figure 2G), whereas plant miRNAs are often found much further, in agreement with the fact that plants usually have longer precursors (83). The GC content of miRNAs exhibits significant variations between species (Figure 2H), with fish miRNAs being notably less GC-rich than those of other species. Finally, we noted a remarkable nucleotide composition bias at the first position of the miRNA with 40% (in mammals) to 60% (in fish) of miRNAs starting with a U nucleotide (Figure 2I).

Feature ranking was then repeated on each set of species separately. While certain structural features such as those relating to the number of base pairings ranked consistently high for all lineages, others, in agreement with the results presented in Table 2, are ranked differently for different species (Table 2 and Supplementary Tables S1). In particular, the distance to and overlap with the terminal loop showed decreased informativity in plants, while the MFE and the number of base pairs in the duplex were more informative in plants than animals.

### Training and evaluation of miRNAs classifiers

**Figure 3.**
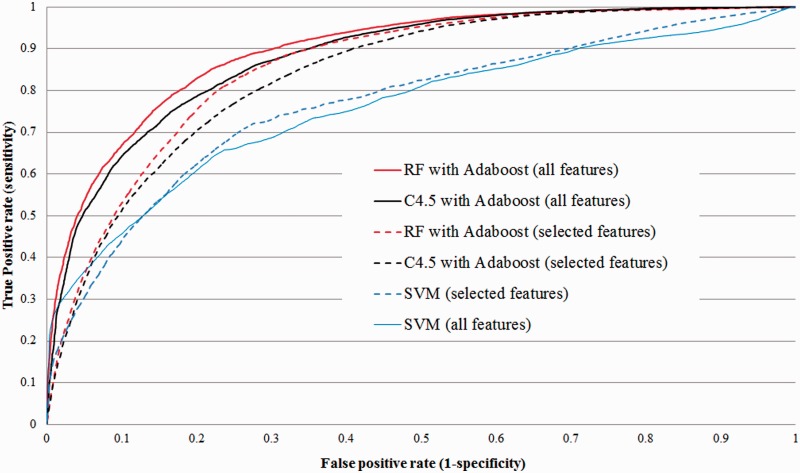
Receiver–operating characteristic curves of classifiers trained on the complete mirBase dataset. See selected features in Table 2.

We first evaluated the classification accuracy of various binary classifiers that, when presented with a candidate miRNA and its pre-miRNA, determine whether the candidate is a positive or negative example. Classifiers were first trained on a balanced data set consisting of 19 823 miRNAs from MirBase (irrespective of species) and the same number of negative instances (randomly selected regions of actual pre-miRNAs, with lengths matched with positive examples; see ‘Materials and Methods’ section) and were evaluated using 10-fold cross-validation (Table 3). Classifiers included a SVM (using a radial basis kernel), a decision tree classifier (C4.5 with Adaboost) and a random forest classifier (with Adaboost). Other learning algorithms were also considered but were found to be less accurate (data not shown); these included RIPPER (84), a feed-forward artificial neural network (85) and a logistic regression classifier (86). Each classifier was trained on either the full set of 100 features or on the subset of 22 best features of the Table 2. The best overall prediction accuracies were obtained by the random forest classifier using all features (Figure 3, Table 3), with an accuracy of 80.6%, an area under the Receiver–operating characteristic curve of 89.2%, and a Matthews correlation coefficient (MCC) of 61.4%. Boosting on C4.5 tree produced similar but slightly inferior results. The SVM classifier trained using all features performed poorly, with an AUC at 75.8%. The SVM’s accuracy improved slightly when restricted to only the 22 most informative features but it remained inferior to that of the random forest classifier.

Knowing that miRNAs properties are different between species, the training and evaluation steps were repeated separately on each of the five clades. We chose to train lineage-specific classifiers using the random forest classifier with no feature selection, as this is the approach that worked best on the full data set. Results are presented in Table 4. Accuracy levels were generally improved as compared with the multilineage classifier, ranging from 81.7 to 86.4%, but with the exception of arthropods, for which the predictions were only 77.7% accurate. For almost all lineages, the accuracy of the lineage-specific predictor (measured using 10-fold cross-validation) is also higher than that of predictors trained on another lineage (Table 5). The inferior performance of the arthropod-specific predictor is likely due to a combination of the small size of the data set, the variability of features within the data set and large diversity of species within the data set.

**Table 1.** Features used in miRdup

Features	Number	Description
miRNA primary sequence		
Single nucleotide frequency	4	Frequency of each nucleotide
Dinucleotide frequency	16	Frequency of each dinucleotide
GC content	1	Frequency of C or G
First/last nucleotide	8	Nucleotide type at the miRNA start and end
Length	1	miRNA length
miRNA–miRNA duplex		
Triplets	32	Frequency of each sequence/structure triplet (42)
Bulges	22	Bulge(s) at positions −4 to +4 nt around start and end of the miRNA. Bulges lengths and number of bulges in the miRNA.
Base pairing	10	Average number of base pairs in duplex and in a sliding window of length 3, 5 and 7 nt. Presence and start position of a perfect 5, 10 and 20 nt base pairs.
Pairs type	3	Percentage of bases forming each type of canonical/wobble base pairs (C-G, A-U, G-U) in the duplex
Loop	2	Percentage of the miRNA overlapping the hairpin loop
MFE	1	MFE of the duplex

To illustrate an important use of miRdup, we used it to reanalyze a set of 1670 miRNA predicted by MiRdeep2 (54) from short-RNA sequencing data in human cancer lines (SRA SRR029124). MiRdup-mammals validated only 755 (45%) of these candidate miRNAs. There are multiple lines of evidence that suggest that the candidates that were rejected by miRdup were indeed MiRdeep2 false positives. First, only 3% of the candidate miRNAs that were rejected by miRdup overlapped annotated miRNAs from MiRbase, whereas this fraction was of 47% among candidate miRNAs that were validated by miRdup. Second, we observe that among the miRNAs predicted by MiRdeep2 and validated by miRdup, a large proportion (46.5%) overlap highly conserved sequences among mammals [based on PhastCons highly conserved elements (87)], whereas this proportion drops to only 19.2% among MiRdeep2 miRNA predictions that were rejected by miRdup. These results suggest that the candidate miRNAs rejected by miRdup are either non-functional, or are atypical, unannotated and poorly conserved miRNAs. Finally, we also reanalyzed the pool of pre-miRNAs and their mature miRNAs predicted and published by the authors of miRdeep (6), and miRdeep2 (54). MiRdup confirmed 89% (201 on 226) and 84% (98 on 117) of the identified miRNAs, respectively.

**Table 2.** Attribute ranking scores evaluated on all miRbase, mammals and plants data sets with Information Gain ranker

Features (Total: 22)	miRbase rank score	Mammals rank score	Plants rank score	Arthropods rank score	Nematodes rank score	Fishes rank score
Average number of paired bases in 3 bp sliding widow	**0.186 [1]**	0.181 [2]	**0.218 [1]**	0.165 [2]	0.190 [5]	0.220 [5]
Length of the longest bulges (% of miRNA length)	0.185 [2]	0.176 [3]	0.203 [5]	0.153 [4]	0.190 [3]	0.193 [3]
Length of the longest bulges (nt)	0.183 [3]	0.175 [4]	0.197 [7]	0.147 [6]	0.196 [2]	0.189 [2]
Average number of paired bases in 5 bp sliding widow	0.174 [5]	0.171 [5]	0.21 [4]	0.163 [3]	0.168 [6]	0.201 [6]
Distance to the terminal loop	0.174 [4]	**0.248 [1]**	0.151 [9]	**0.190 [1]**	**0.306 [1]**	**0.274 [1]**
Number of paired bases in the miRNA–miRNA* duplex	0.165 [6]	0.151 [8]	0.213 [3]	0.137 [7]	0.182 [4]	0.188 [4]
Average number of paired bases in 7 bp sliding widow	0.159 [7]	0.156 [7]	0.2 [6]	0.136 [8]	0.146 [7]	0.181 [7]
Length of miRNA overlap within the hairpin loop	0.147 [8]	0.167 [6]	0.107 [14]	0.115 [9]	0.145 [8]	0.150 [8]
MFE of the duplex	0.137 [9]	0.112 [10]	0.214 [2]	0.162 [5]	0.102 [12]	0.196 [12]
Percentage of GC base pairs in the duplex	0.122 [10]	0.09 [14]	0.102 [15]	0.060 [16]	0.059 [17]	0.068 [17]
Percentage of AU base pairs in the duplex	0.118 [11]	0.068 [18]	0.083 [19]	0.027 [22]	0.058 [18]	0.046 [18]
Triplet U	0.117 [12]	0.114 [9]	0.124 [10]	0.106 [10]	0.107 [11]	0.128 [11]
Distance to the start of the hairpin	0.112 [13]	0.094 [13]	0.155 [8]	0.077 [14]	0.144 [9]	0.107 [9]
Triplet A	0.111 [14]	0.099 [12]	0.113 [11]	0.085 [12]	0.126 [10]	0.114 [10]
miRNA included in loop (yes/no)	0.107 [15]	0.105 [11]	0.076 [20]	0.058 [17]	0.088 [14]	0.067 [14]
Triplet C	0.082 [16]	0.074 [17]	0.09 [17]	0.063 [15]	0.091 [13]	0.101 [13]
Percentage of GU base pairs in the duplex	0.074 [17]	0.076 [16]	0.084 [18]	0.034 [20]	0.045 [19]	0.055 [19]
Triplet G	0.068 [18]	0.08 [15]	0.069 [21]	0.082 [13]	0.082 [15]	0.110 [15]
Position of the first 5 nt bulge-free region	0.066 [19]	0.059 [19]	0.098 [16]	0.103 [11]	0.076 [16]	0.124 [16]
Triplet G	0.059 [20]	0.029 [22]	0.058 [22]	0.027 [21]	0.022 [21]	0.040 [21]
Maximum length without bulges (nt)	0.058 [22]	0.05 [21]	0.112 [12]	0.038 [19]	0.037 [20]	0.056 [20]
Maximum length without bulges (% of the miRNA length)	0.058 [21]	0.051 [20]	0.11 [13]	0.049 [18]	0.033 [22]	0.063 [22]

Scores are based on the information gain between the attribute and the class (67). Best score is bold. Features with substantially different scores (>0.05) in mammals versus plants are underlined. Full ranking values are in Supplementary Tables S1 for miRbase, mammals and plants.

### Prediction of a miRNA position within a pre-miRNA

**Figure 4.**
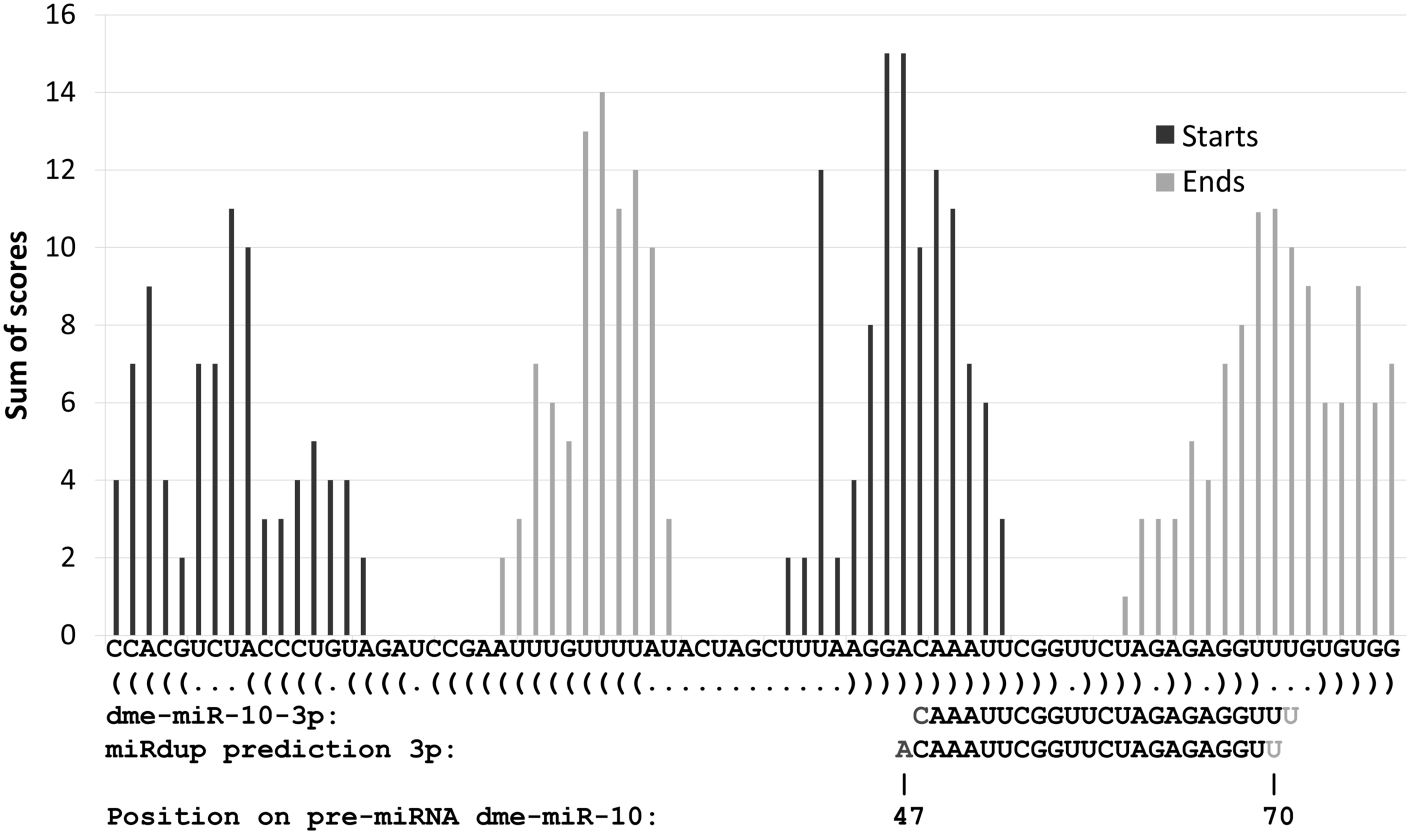
Example of miRdup prediction on the *Drosophila melanogaster* dme-mir-10 pre-miRNA. The actual miRNA predicted in 3p extends from position 48 to 71 of the pre-miRNA, and the predicted miRNA in 5p, the miRNA*, is CUACCCUGUAGAUCCGAAUUUGUU. Dark columns show the score assigned by miRdup-arthropods to each possible starting position (summed over all possible lengths), and in gray columns are scores of each possible ending position. MiRdup predicts that the miRNA extends from positions 47 to 70, off by one position compared with the true miRNA.

MiRdup can be used to predict the most likely miRNA duplex location, i.e. the most likely miRNA in 5 prime (5p) and 3 prime (3p), within a given pre-miRNA. Given a pre-miRNA sequence and a trained classifier for the binary decision problem, miRdup computes prediction scores for every possible combination of miRNA length (16–30 nt) and starting position and then identifies the pair of starting and ending positions, located within 16–30 nt of each other, for which the total evidence is highest (see ‘Materials and Methods’ section). We finally return the predicted miRNA and its miRNA*. Figure 4 shows an example of the prediction made for a typical pre-miRNA, *Drosophila melanogaster*’s dme-mir-10.

**Figure 5.**
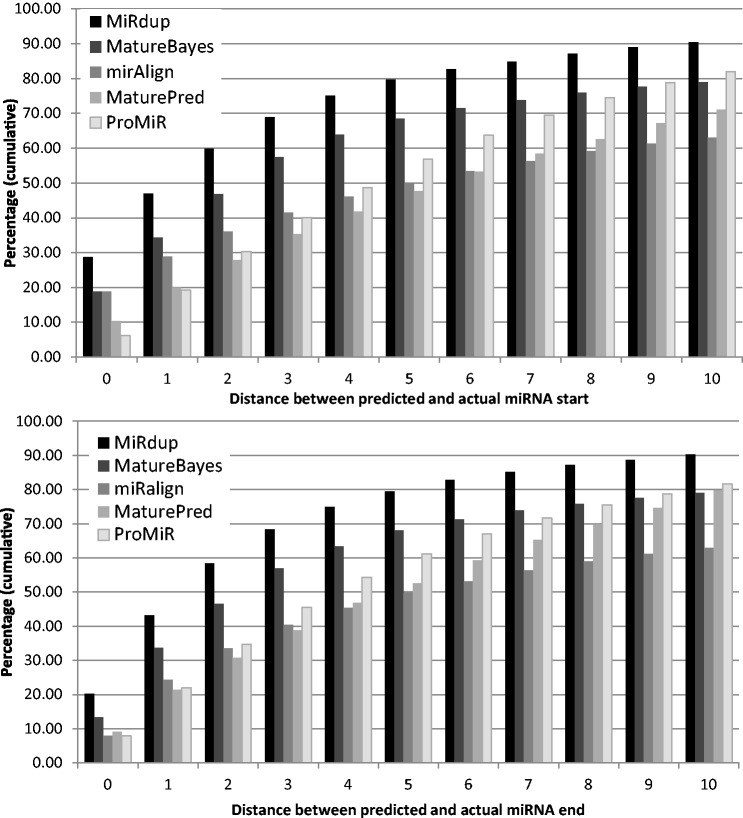
Cumulative distribution of the minimum distance between the true and predicted miRNAs or miRNAs* starts (up) and ends (down), i.e. the proportion of cases where the prediction is within x bases of the true start/end positions. Multilineage miRdup predictions are compared with MatureBayes (57), MiRalign (61), MaturePred (63) and PromiR1 (40) for all experimentally validated pre-miRNAs from miRbase, except for MaturePred, where our analysis was limited to only 2400 miRNAs submitted owing to web server constraints. For MatureBayes and Promir, a small number of queries were rejected by the web server and were thus excluded from the results. We only show distances of up to 10 nt, but in some rare cases, errors are substantially larger (up to 250 nt). Results for lineage-specific miRdup compared with MatureBayes for mammals, arthropods, nematods, fish and plants are shown in Supplementary Figure S1.

To estimate the accuracy of miRdup at locating miRNAs within pre-miRNAs, we calculated the minimum distance between the true and predicted miRNA/miRNA*, for both the start and end positions (Figure 5 and Supplementary Figure S1). When trained and evaluated on data from all five lineages combined, miRdup made perfect predictions of start and end positions in 28.7% and 20.18% of the cases, respectively, and was within 3 nt in 68.9% and 68.3% of cases, respectively. This is significantly better than MatureBayes, miRalign and ProMir 1, the only miRNA predictors we were able to compare with. When evaluated on the same data set, MatureBayes yields only 18.8% and 13.3% exact miRNA duplex start and end position predictions, while MiRalign yields 18.8% and 7.9%, MaturePred 10.34% and 9.14% and ProMir1 6.13% and 8.01%. The results also indicate that ∼10% of the predictions are off by >10 nt with miRdup, versus ∼20% for the best of the competitors (Figure 5).

**Table 3.** Results of various classifiers trained on all features of miRbase (all lineages) evaluated using 10-fold cross-validation

Classifier	Correctly classified instances (out of 39 646)	Sensitivity	Specificity	Accuracy	MCC	AUC
Random forest with AdaBoost	31 940	0.863	0.748	0.806	0.614	0.892
C4.5 decision tree with AdaBoost	31 317	0.809	0.771	0.79	0.58	0.875
SVM with radial basis kernel	25 878	0.344	0.962	0.653	0.385	0.653

**Table 4.** Prediction accuracy of lineage-specific miRdup predictors (random forest with Adaboost, evaluated using 10-fold cross-validation)

Classifier	Number of instances	Correctly classified instances	Sensitivity	Specificity	ACC	MCC	AUC
Mammals	13 918	11 415	0.868	0.772	0.82	0.642	0.897
Plants	9464	7734	0.866	0.768	0.817	0.636	0.904
Nematods	2174	1789	0.882	0.764	0.823	0.649	0.898
Arthropods	5240	4071	0.833	0.721	0.777	0.557	0.857
Fish	1530	1323	0.905	0.824	0.864	0.731	0.918

**Table 5.** Accuracy of lineage-specific and non-lineage-specific miRdup predictors (rows) for the prediction of miRNAs from each lineage (columns)

Test set |Training set	miRbase	Nematods	Arthropods	Fish	Mammals	Plants
miRbase	**0.806**	0.818	**0.807**	0.852	0.808	0.790
Nematods	0.74	0.823	0.755	0.82	0.768	0.618
Arthropods	0.768	0.812	0.777	0.806	0.765	0.712
Fish	0.716	0.808	0.72	**0.864**	0.741	0.606
Mammals	0.793	**0.834**	0.766	0.846	**0.820**	0.655
Plants	0.700	0.662	0.644	0.681	0.645	**0.817**

The highest accuracy for each column is in bold. For cases where a predictor is applied to data from the lineage it is trained on, the numbers reported are obtained by 10-fold cross-validation.

Results obtained using the appropriate lineage-specific version of miRdup generally improve on the multilineage predictor, with 25.9–34.0% (respectively 20.7–24.6%) of start (respectively end) positions predicted exactly correctly (Supplementary Figure S1). Again, fish miRNAs stand out as being the easiest to predict, with 51.5% (respectively 29.6%) of start (respectively end) positions correctly predicted.

### The miRdup program

**Figure 6.**
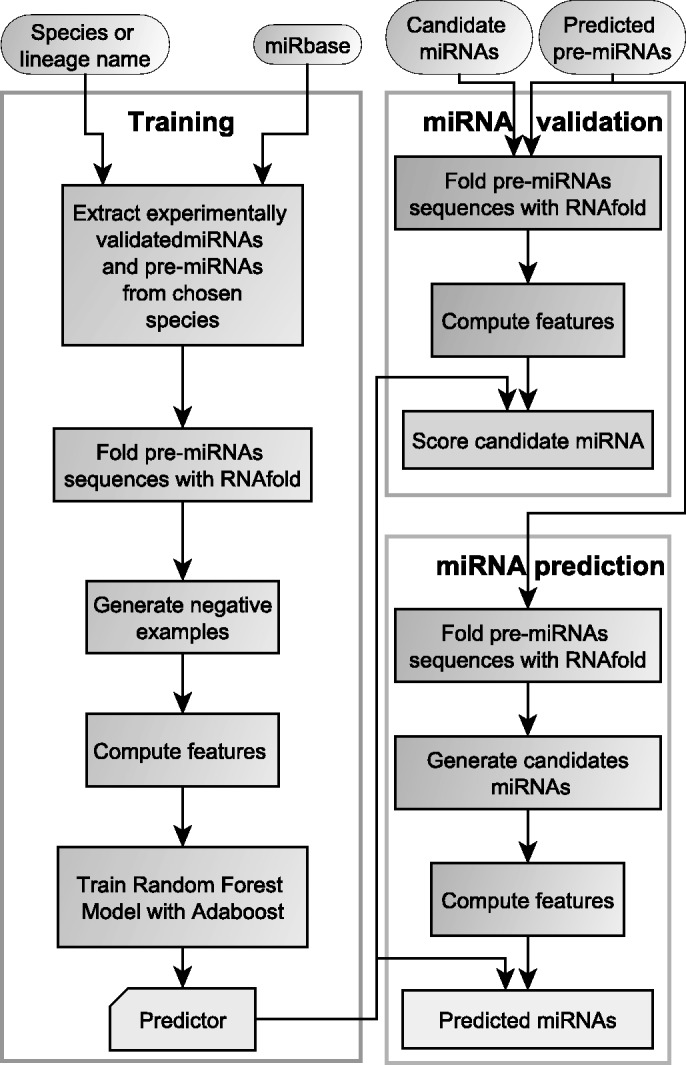
Workflow of the miRdup algorithm.

MiRdup is distributed as a java program making use of libraries from the Weka (67) and ViennaRNA (66) packages. The workflow is schematized in Figure 6. MiRdup can either be trained on a user-provided dataset of known miRNAs and pre-miRNAs, or can automatically download the latest version of mirBase and be trained on all of it or on a lineage-specific subset. For example, if ‘ruminantia’ is specified as clade of interest, the predictor will be trained only on *Bos taurus* and *Ovis aries*, which are (currently) the only two species present in miRbase in this clade. The set of negative examples is constructed on the fly by randomizing the position of miRNAs on the pre-miRNA. A MFE secondary structure is obtained for each pre-miRNAs, and features are calculated. Finally, the random forest predictor is trained. MiRdup can be run in two modes. In the first, miRdup takes as input a pre-miRNA sequence (with or without predicted secondary structure) and a candidate miRNA position, and assigns a score reflecting the likelihood that the candidate is a real miRNA. In the second case, miRdup evaluates every possible combination of miRNA position and length, and reports the most likely pair.

Thanks to its relative simplicity, miRdup is fast. On a computer with a single 2.93 GHz CPU, the training phase on the complete mirBase database requires <80 min, and the miRNA prediction phase takes ∼10 s for a given pre-miRNA of 100 nt.

## CONCLUSIONS

Although the structural properties of pre-miRNAs are well characterized (88) and have largely been exploited for their predictions (89), the sequence and structure properties that allow Dicer to recognize the exact position of the mature miRNA remains poorly understood (90). For this reason, computational approaches for the identification of miRNAs within pre-miRNA are rare and relatively inaccurate. Such predictors are, however, of great importance. First, working hand in hand with pre-miRNA predictors, they are essential for the *de novo* computational miRNA annotation of new genomes. Second, they play an important role even for miRNA annotation projects that have the benefit of short-RNA sequencing data. Indeed, from our experience, the classical approach of identifying likely miRNAs by retaining only reads that map to a genomic regions with strong pre-miRNA potential (as predicted by miPred (44) or HHMMiR (47), for example) still yields tens of thousands predictions. Considering only candidates overlapping pre-miRNAs predicted by more than one tool can reduce this number, but the consequences on sensitivity and specificity are hard to quantify. A more reasonable number of predictions can be obtained by more recent tools such as miRDeep (6,54), although even it often produced unlikely miRNA predictions. MiRdup then offers the opportunity to discard these likely false positives while retaining a high sensitivity.

MiRdup is a flexible, accurate, fast and user-friendly tool for the localization of mature miRNAs in pre-miRNA. It complements a wide array of computational tools that aim to identify pre-miRNAs and should be used as a posttreatment of predicted hairpins or to validate the miRNA function of short RNA reads mapped to a reference genome. MiRdup’s speed and flexibility let it to be trained on data from specific lineages, which allows it to take advantage of species-specific miRNA properties. Because it is automatically trained on the latest version of mirBase, it remains up-to-date and can take advantage of increasingly large and accurate sets of miRNA annotations. The multilineage version of MiRdup outperforms the only other miRNA predictor available for download, matureBayes (62). The lineage-specific version is even more accurate, as it is able to take advantage of features such as the presence of a uracyl at the first position of the vast majority of fish miRNAs, or the increased stability of the miRNA–miRNA* duplex in plants.

## SUPPLEMENTARY DATA

Supplementary Data are available at NAR Online: Supplementary Table 1 and Supplementary Figure 1.

## FUNDING

Fonds Québécois pour la Recherche sur la Nature et les Technologies, B2 research grant (to M.L.). Funding for open access charge: Natural Sciences and Engineering Research Council of Canada.

*Conflict of interest statement*. None declared.

## Supplementary Material

Supplementary Data

## References

[1] Ambros V (1989). A hierarchy of regulatory genes controls a larva-to-adult developmental switch in *C. elegans*. Cell.

[2] Ruvkun G (2001). Molecular biology. Glimpses of a tiny RNA world. Science.

[3] Swami M (2010). Small RNAs: an epigenetic silencing influence. Nat. Rev. Genet..

[4] Lee Y, Kim M, Han J, Yeom KH, Lee S, Baek SH, Kim VN (2004). MicroRNA genes are transcribed by RNA polymerase II. EMBO J..

[5] Cuperus JT, Fahlgren N, Carrington JC (2011). Evolution and functional diversification of MIRNA genes. Plant Cell.

[6] Friedlander MR, Chen W, Adamidi C, Maaskola J, Einspanier R, Knespel S, Rajewsky N (2008). Discovering microRNAs from deep sequencing data using miRDeep. Nat. Biotechnol..

[7] Schwarz DS, Hutvagner G, Du T, Xu Z, Aronin N, Zamore PD (2003). Asymmetry in the assembly of the RNAi enzyme complex. Cell.

[8] Lagos-Quintana M, Rauhut R, Yalcin A, Meyer J, Lendeckel W, Tuschl T (2002). Identification of tissue-specific microRNAs from mouse. Curr. Biol..

[9] Lim LP, Lau NC, Garrett-Engele P, Grimson A, Schelter JM, Castle J, Bartel DP, Linsley PS, Johnson JM (2005). Microarray analysis shows that some microRNAs downregulate large numbers of target mRNAs. Nature.

[10] Carrington JC, Ambros V (2003). Role of microRNAs in plant and animal development : developmental timing. Science.

[11] Cuellar TL, McManus MT (2005). MicroRNAs and endocrine biology. J. Endocrinol..

[12] Suh MR, Lee Y, Kim JY, Kim SK, Moon SH, Lee JY, Cha KY, Chung HM, Yoon HS, Moon SY (2004). Human embryonic stem cells express a unique set of microRNAs. Dev. Biol..

[13] Williams AH, Liu N, van Rooij E, Olson EN (2009). MicroRNA control of muscle development and disease. Curr. Opin. Cell Biol..

[14] Chen JF, Mandel EM, Thomson JM, Wu Q, Callis TE, Hammond SM, Conlon FL, Wang DZ (2006). The role of microRNA-1 and microRNA-133 in skeletal muscle proliferation and differentiation. Nat. Genet..

[15] Shivdasani RA (2006). MicroRNAs: regulators of gene expression and cell differentiation. Blood.

[16] Ambros V (2004). The functions of animal microRNAs. Nature.

[17] Brennecke J, Hipfner DR, Stark A, Russell RB, Cohen SM (2003). Bantam encodes a developmentally regulated microRNA that controls cell proliferation and regulates the proapoptotic gene hid in *Drosophila*. Cell.

[18] Poy MN, Eliasson L, Krutzfeldt J, Kuwajima S, Ma X, Macdonald PE, Pfeffer S, Tuschl T, Rajewsky N, Rorsman P (2004). A pancreatic islet-specific microRNA regulates insulin secretion. Nature.

[19] Wilfred BR, Wang WX, Nelson PT (2007). Energizing miRNA research: A review of the role of miRNAs in lipid metabolism, with a prediction that miR-103/107 regulates human metabolic pathways. Mol. Genet. Metab..

[20] Miska EA, Alvarez-Saavedra E, Abbott AL, Lau NC, Hellman AB, McGonagle SM, Bartel DP, Ambros VR, Horvitz HR (2007). Most *Caenorhabditis* elegans microRNAs are individually not essential for development or viability. PLoS Genet..

[21] Clop A, Marcq F, Takeda H, Pirottin D, Tordoir X, Bibe B, Bouix J, Caiment F, Elsen JM, Eychenne F (2006). A mutation creating a potential illegitimate microRNA target site in the myostatin gene affects muscularity in sheep. Nat. Genet..

[22] Castanotto D, Rossi JJ (2009). The promises and pitfalls of RNA-interference-based therapeutics. Nature.

[23] Cooper TA, Wan L, Dreyfuss G (2009). RNA and disease. Cell.

[24] Murchison EP, Stein P, Xuan Z, Pan H, Zhang MQ, Schultz RM, Hannon GJ (2007). Critical roles for Dicer in the female germline. Genes Dev..

[25] Osada H, Takahashi T (2007). MicroRNAs in biological processes and carcinogenesis. Carcinogenesis.

[26] Fujii H, Chiou TJ, Lin SI, Aung K, Zhu JK (2005). A miRNA involved in phosphate-starvation response in *Arabidopsis*. Curr. Biol..

[27] Guy CL (1990). Cold accelimation and freezing stress tolerance: role of protein metabolism. Annu. Rev. Plant Biol..

[28] Jones-Rhoades MW, Bartel DP (2004). Computational identification of plant microRNAs and their targets, including a stress-induced miRNA. Mol. Cell.

[29] Saqib M, Zorb C, Schubert S (2008). Silicon-mediated improvement in the salt resistance of wheat (Triticum aestivum) results from increased sodium exclusion and resistance to oxidative stress. Funct. Plant Biol..

[30] Sunkar R, Kapoor A, Zhu JK (2006). Posttranscriptional induction of two Cu/Zn superoxide dismutase genes in Arabidopsis is mediated by downregulation of miR398 and important for oxidative stress tolerance. Plant Cell.

[31] Van Rooij E, Sutherland LB, Liu N, Williams AH, McAnally J, Gerard RD, Richardson JA, Olson EN (2006). A signature pattern of stress-responsive microRNAs that can evoke cardiac hypertrophy and heart failure. Proc. Natl Acad. Sci. USA.

[32] Nelson JA (2007). Small RNAs and large DNA viruses. N. Engl. J. Med..

[33] Pfeffer S, Sewer A, Lagos-Quintana M, Sheridan R, Sander C, Grasser FA, van Dyk LF, Ho CK, Shuman S, Chien M (2005). Identification of microRNAs of the herpesvirus family. Nat. Methods.

[34] Sarnow P, Jopling CL, Norman KL, Schutz S, Wehner KA (2006). MicroRNAs: expression, avoidance and subversion by vertebrate viruses. Nat. Rev. Microbiol..

[35] Berezikov E, Cuppen E, Plasterk RH (2006). Approaches to microRNA discovery. Nat. Genet..

[36] Li L, Xu J, Yang D, Tan X, Wang H (2010). Computational approaches for microRNA studies: a review. Mamm. Genome.

[37] Schulte JH, Marschall T, Martin M, Rosenstiel P, Mestdagh P, Schlierf S, Thor T, Vandesompele J, Eggert A, Schreiber S (2010). Deep sequencing reveals differential expression of microRNAs in favorable versus unfavorable neuroblastoma. Nucleic Acids Res..

[38] Sunkar R, Zhou X, Zheng Y, Zhang W, Zhu JK (2008). Identification of novel and candidate miRNAs in rice by high throughput sequencing. BMC Plant Biol..

[39] Grey F, Antoniewicz A, Allen E, Saugstad J, McShea A, Carrington JC, Nelson J (2005). Identification and characterization of human cytomegalovirus-encoded microRNAs. J. Virol..

[40] Nam JW, Shin KR, Han JJ, Lee Y, Kim VN, Zhang BT (2005). Human microRNA prediction through a probabilistic co-learning model of sequence and structure. Nucleic Acids Res..

[41] Nam JW, Kim J, Kim SK, Zhang BT (2006). ProMiR II: a web server for the probabilistic prediction of clustered, nonclustered, conserved and nonconserved microRNAs. Nucleic Acids Res..

[42] Xue CH, Li F, He T, Liu GP, Li YD, Zhang XG (2005). Classification of real and pseudo microRNA precursors using local structure-sequence features and support vector machine. BMC Bioinformatics.

[43] Sewer A, Paul N, Landgraf P, Aravin A, Pfeffer S, Brownstein MJ, Tuschl T, van Nimwegen E, Zavolan M (2005). Identification of clustered microRNAs using an ab initio prediction method. BMC Bioinformatics.

[44] Jiang P, Wu H, Wang W, Ma W, Sun X, Lu Z (2007). MiPred: classification of real and pseudo microRNA precursors using random forest prediction model with combined features. Nucleic Acids Res..

[45] Oulas A, Boutla A, Gkirtzou K, Reczko M, Kalantidis K, Poirazi P (2009). Prediction of novel microRNA genes in cancer-associated genomic regionsa combined computational and experimental approach. Nucleic Acids Res..

[46] Batuwita R, Palade V (2009). microPred: effective classification of pre-miRNAs for human miRNA gene prediction. Bioinformatics.

[47] Kadri S, Hinman V, Benos PV (2009). HHMMiR: efficient de novo prediction of microRNAs using hierarchical hidden Markov models. BMC Bioinformatics.

[48] Thieme CJ, Gramzow L, Lobbes D, TheiBen G (2011). SplamiR: prediction of spliced miRNAs in plants. Bioinformatics.

[49] Bonnet E, Wuyts J, Rouzé P, Van de Peer Y (2004). Detection of 91 potential conserved plant microRNAs in Arabidopsis thaliana and Oryza sativa identifies important target genes. Proc. Natl Acad. Sci. USA.

[50] Ding JD, Zhou SG, Guan JH (2010). MiRenSVM: towards better prediction of microRNA precursors using an ensemble SVM classifier with multi-loop features. BMC Bioinformatics.

[51] Griffiths-Jones S, Grocock RJ, van Dongen S, Bateman A, Enright AJ (2006). miRBase: microRNA sequences, targets and gene nomenclature. Nucleic Acids Res..

[52] Griffiths-Jones S, Saini HK, van Dongen S, Enright AJ (2008). miRBase: tools for microRNA genomics. Nucleic Acids Res..

[53] Lindow M, Jacobsen A, Nygaard S, Mang Y, Krogh A (2007). Intragenomic matching reveals a huge potential for miRNA-mediated regulation in plants. PLoS Comput. Biol..

[54] Friedlander MR, Mackowiak SD, Li N, Chen W, Rajewsky N (2012). miRDeep2 accurately identifies known and hundreds of novel microRNA genes in seven animal clades. Nucleic Acids Res..

[55] An J, Lai J, Lehman ML, Nelson CC (2013). miRDeep*: an integrated application tool for miRNA identification from RNA sequencing data. Nucleic Acids Res..

[56] Mathelier A, Carbone A (2010). MIReNA: finding microRNAs with high accuracy and no learning at genome scale and from deep sequencing data. Bioinformatics.

[57] Hackenberg M, Rodriguez-Ezpeleta N, Aransay AM (2011). miRanalyzer: an update on the detection and analysis of microRNAs in high-throughput sequencing experiments. Nucleic Acids Res..

[58] Hackenberg M, Sturm M, Langenberger D, Falcon-Perez JM, Aransay AM (2009). miRanalyzer: a microRNA detection and analysis tool for next-generation sequencing experiments. Nucleic Acids Res..

[59] Fahlgren N, Jogdeo S, Kasschau KD, Sullivan CM, Chapman EJ, Laubinger S, Smith LM, Dasenko M, Givan SA, Weigel D (2010). MicroRNA gene evolution in *Arabidopsis lyrata* and *Arabidopsis thaliana*. Plant cell.

[60] Breakfield NW, Corcoran DL, Petricka JJ, Shen J, Sae-Seaw J, Rubio-Somoza I, Weigel D, Ohler U, Benfey PN (2012). High-resolution experimental and computational profiling of tissue-specific known and novel miRNAs in *Arabidopsis*. Genome Res..

[61] Wang X, Zhang J, Li F, Gu J, He T, Zhang X, Li Y (2005). MicroRNA identification based on sequence and structure alignment. Bioinformatics.

[62] Gkirtzou K, Tsamardinos I, Tsakalides P, Poirazi P (2010). MatureBayes: a probabilistic algorithm for identifying the mature miRNA within novel precursors. PLoS One.

[63] Xuan P, Guo MZ, Huang YC, Li WB, Huang YF (2011). MaturePred: efficient identification of microRNAs within novel plant pre-miRNAs. PLoS One.

[64] He C, Li YX, Zhang G, Gu Z, Yang R, Li J, Lu ZJ, Zhou ZH, Zhang C, Wang J (2012). MiRmat: mature microRNA sequence prediction. PLoS One.

[65] Yang JS, Phillips MD, Betel D, Mu P, Ventura A, Siepel AC, Chen KC, Lai EC (2011). Widespread regulatory activity of vertebrate microRNA* species. RNA.

[66] Hofacker IL, Fontana W, Stadler PF, Bonhoeffer LS, Tacker M, Schuster P (1994). Fast folding and comparison of RNA secondary structures. Monatsh. Chem..

[67] Hall M, Frank E, Holmes G, Pfahringer B, Reutemann P, Witten IH (2009). The WEKA data mining software: an update. ACM SIGKDD Explor. Newsl..

[68] Dash M, Liu H (1997). Feature selection for classification. Intell. Data Anal..

[69] Hong SJ (1997). Use of contextual information for feature ranking and discretization. IEEE Trans. Knowl. Data Eng..

[70] Robnik-Sikonja M, Kononenko I (2003). Theoretical and empirical analysis of ReliefF and RReliefF. Mach. Learn..

[71] Quinlan JR (1986). Induction of decision trees. Mach. Learn..

[72] Shannon CE, Weaver W, Blahut RE, Hajek B (1949). The Mathematical Theory of Communication.

[73] Breiman L (2001). Random forests. Mach. Learn..

[74] Freund Y, Schapire R (1997). A decision-theoretic generalization of on-line learning and an application to boosting. Journal of Computer and System Sciences.

[75] Osman IH, Kelly JP (1996). Meta-heuristics: Theory and Applications.

[76] Cortes C, Vapnik V (1995). Support-vector networks. Mach. Learn..

[77] Chang CC, Lin CJ (2001). LIBSVM: A Library for Support Vector Machines. [Computer Program].

[78] Quinlan JR (1993). C4.5: Programs for Machine Learning.

[79] Matthews BW (1975). Comparison of the predicted and observed secondary structure of T4 phage lysozyme. Biochim. Biophys. Acta.

[80] Zhou J, Foster DP, Stine RA, Ungar LH (2006). Streamwise feature selection. J. Mach. Learn. Res..

[81] Robnik-Sikonja M (2004). Improving random forests. Lect. Notes Comput. Sc..

[82] Guyon I, Weston J, Barnhill S, Vapnik V (2002). Gene selection for cancer classification using support vector machines. Mach. Learn..

[83] Zhang BH, Pan XP, Cox SB, Cobb GP, Anderson TA (2006). Evidence that miRNAs are different from other RNAs. Cell. Mol. Life Sci..

[84] Cohen WW (1995). Proceedings of the Twelfth International Conference on Machine Learning.

[85] Collobert R, Bengio S (2004). Proceedings of the twenty-first international conference on Machine learning.

[86] Le Cessie S, Van Houwelingen J (1992). Ridge estimators in logistic regression. Appl. Stat..

[87] Siepel A, Bejerano G, Pedersen JS, Hinrichs AS, Hou M, Rosenbloom K, Clawson H, Spieth J, Hillier LW, Richards S (2005). Evolutionarily conserved elements in vertebrate, insect, worm, and yeast genomes. Genome Res..

[88] Krol J, Sobczak K, Wilczynska U, Drath M, Jasinska A, Kaczynska D, Krzyzosiak WJ (2004). Structural features of MicroRNA (miRNA) precursors and their relevance to miRNA biogenesis and small interfering RNA/Short hairpin RNA design. J. Biol. Chem..

[89] Mendes ND, Freitas AT, Sagot MF (2009). Current tools for the identification of miRNA genes and their targets. Nucleic Acids Res..

[90] Park JE, Heo I, Tian Y, Simanshu DK, Chang H, Jee D, Patel DJ, Kim VN (2011). Dicer recognizes the 5′ end of RNA for efficient and accurate processing. Nature.

