# Supplementary material for: Computational prediction of the localization of microRNAs within their pre-miRNA
Source: Nucleic Acids Res. 2013 Jun 8;41(15):7200–11. doi: 10.1093/nar/gkt466 (PMC3753617; doi:10.1093/nar/gkt466)
Supplement: Supplementary Data [file supp_41_15_7200__index.html]

Computational prediction of the localization of microRNAs within their pre-miRNA — Computational prediction of the localization of microRNAs within their pre-miRNA — Supplementary Data 

# Computational prediction of the localization of microRNAs within their pre-miRNA

## Supplementary Data

files

**Files in this Data Supplement:**

- Supplementary Data - pdf file
