# Supplementary material for: Computational prediction of the localization of microRNAs within their pre-miRNA
Source: Nucleic Acids Res. 2013 Jun 8;41(15):7200–11. doi: 10.1093/nar/gkt466 (PMC3753617; doi:10.1093/nar/gkt466)
Supplement: Supplementary Data [file supp_gkt466_nar-00624-n-2013-File008.pdf]

Supplementary data

Supplementary tables 1: Attribute rankings

Attribute ranking output for miRbase

Search Method: Attribute ranking.

Attribute Evaluator (supervised, Class (nominal): 101 Class): Information Gain Ranking Filter

Rank scores	ID	Features
0.183809	40	Average number of paired bases in a sliding widow of 3nt along the miRNA
0.178933	59	Length of the biggest bulges in percentage of the miRNA length
0.177118	58	Length of the biggest bulges in nucleotides
0.174022	39	Average number of paired bases in a sliding widow of 5nt along the miRNA
0.173801	34	Distance of the miRNA from the terminal loop of the hairpin
0.163464	7	Bases pairs in the duplex of the miRNA and its complementarity region
0.159278	38	Average number of paired bases in a sliding widow of 7nt along the miRNA
0.143582	37	Length of the miRNA which overlap in the hairpin loop
0.127442	2	Minimum free energy of the duplex
0.114706	63	Triplet U...
0.108587	60	Triplet A...
0.105129	35	Distance of the start of miRNA from the start of the hairpin
0.100427	93	Percentage of GC base pairs in the duplex
0.094015	92	Percentage of AU base pairs in the duplex
0.092867	36	miRNA included in loop
0.084723	94	Percentage of GU base pairs in the duplex
0.079484	61	Triplet C...
0.072825	62	Triplet G...
0.070982	13	Start of perfect 5 nt base pair in the miRNA
0.059999	4	Maximum length without bulges
0.059992	5	Maximum length without bulges in percentage of the miRNA length
0.042843	45	Bulge at start position 2
0.038907	95	nucleotide at start position 0
0.037558	47	Bulge at start position 3
0.0335	78	Triplet G(((
0.031529	11	Start of perfect 10 nt base pair in the miRNA
0.027518	6	Length without bulges from miRNA start
0.027212	55	Bulge at end position -3
0.024292	10	PresenceOfPerfect10MerBasePair
0.023319	12	PresenceOfPerfect5MerBasePair
0.021186	67	Triplet U(..
0.018154	76	Triplet A(((
0.017903	43	Bulge at position 1
0.017436	3	Percentage of GC content
0.015656	79	Triplet U(((
0.015532	66	Triplet G(..
0.013398	64	Triplet A(..
0.013038	53	Bulge at end position -2
0.011222	44	Bulge at start position -2
0.011043	77	Triplet C(((
0.00878	42	Bulge at start position -1
0.008604	97	nucleotide at start position +1
0.008093	16	Percentage of G
0.00776	9	Start of perfect 20 nt base pair in the miRNA
0.007667	65	Triplet C(..
0.007279	8	PresenceOfPerfect20MerBasePair
0.007188	56	Bulge at end position +4
0.007093	49	Bulge at end position 0
0.006354	51	Bulge at end position -1
0.006253	26	Percentage of dinucleotides AG
0.00574	22	Percentage of dinucleotides AU
0.005649	96	nucleotide at start position -1
0.005625	71	U((.
0.004176	23	Percentage of dinucleotides UU

0.004003	28	Percentage of dinucleotides GG
0.003838	57	Number of bulges
0.003728	27	Percentage of dinucleotides UG
0.003705	18	Percentage of dinucleotides AA
0.003683	99	nucleotide at end position -1
0.003494	14	Percentage of dinucleotides A
0.003112	20	Percentage of dinucleotides GA
0.003001	15	Percentage of U
0.002952	69	Triplet C((.
0.002788	32	Percentage of dinucleotides GC
0.002381	87	Triplet U..(
0.002362	48	Bulge at start position -4
0.002161	19	Percentage of dinucleotides UA
0.00194	46	Bulge at start position -3
0.001851	70	Triplet G((.
0.001651	68	Triplet A((.
0.001602	85	Triplet C..(
0.001488	86	Triplet G..(
0.001233	41	Bulge at start position 0
0.0012	31	Percentage of dinucleotides UC
0.001184	84	Triplet A..(
0.00115	74	Triplet G.((
0.001109	72	Triplet A.((
0.001057	82	Triplet G.(.
0.001009	81	Triplet C.(.
0.000973	100	nucleotide at end position +1
0.000869	17	Percentage of C
0.000819	54	Bulge at end position +3
0.000743	83	Triplet U.(.
0.000637	88	Triplet A(.(
0.000523	25	Percentage of dinucleotides CU
0.000421	24	Percentage of dinucleotides GU
0.000412	50	Bulge at end position +1
0.000376	75	Triplet U.((
0.000369	52	Bulge at end position +2
0.000306	80	Triplet A.(.
0.000271	89	Triplet C(.(
0.000208	98	nucleotide at end position 0
0	1	Length
0	21	Percentage of dinucleotides CA
0	29	Percentage of dinucleotides CG
0	30	Percentage of dinucleotides AC
0	33	Percentage of dinucleotides CC
0	73	Triplet C.((
0	90	Triplet G(.(
0	91	Triplet U(.(

Attribute ranking output for Mammals

**Rank scores**	**ID**	**Features**
0.247652	34	Distance of the miRNA from the terminal loop of the hairpin
0.1811	40	Average number of paired bases in a sliding widow of 3nt along the miRNA
0.176017	59	Length of the biggest bulges in percentage of the miRNA length
0.175173	58	Length of the biggest bulges in nucleotides
0.171369	39	Average number of paired bases in a sliding widow of 5nt along the miRNA
0.166916	37	Length of the miRNA which overlap in the hairpin loop
0.156208	38	Average number of paired bases in a sliding widow of 7nt along the miRNA
0.151227	7	Bases pairs in the duplex of the miRNA and its complementarity region
0.114016	63	Triplet U...
0.112425	2	Minimum free energy of the duplex
0.10498	36	miRNA included in loop
0.099441	60	Triplet A...
0.094377	35	Distance of the start of miRNA from the start of the hairpin

0.090307	93	Percentage of GC base pairs in the duplex
0.079723	62	Triplet G...
0.076208	94	Percentage of GU base pairs in the duplex
0.073829	61	Triplet C...
0.068416	92	Percentage of AU base pairs in the duplex
0.058998	13	Start of perfect 5 nt base pair in the miRNA
0.050537	5	Maximum length without bulges in percentage of the miRNA length
0.04952	4	Maximum length without bulges
0.037305	47	Bulge at start position 3
0.030906	95	nucleotide at start position 0
0.029182	45	Bulge at start position 2
0.028684	78	Triplet G(((
0.024654	11	Start of perfect 10 nt base pair in the miRNA
0.022153	55	Bulge at end position -3
0.020967	76	Triplet A(((
0.019976	66	Triplet G(..
0.019604	10	PresenceOfPerfect10MerBasePair
0.019576	12	PresenceOfPerfect5MerBasePair
0.019012	67	Triplet U(..
0.018733	6	Length without bulges from miRNA start
0.016738	79	Triplet U(((
0.01174	56	Bulge at end position +4
0.011673	43	Bulge at position 1
0.011558	53	Bulge at end position -2
0.011164	64	Triplet A(..
0.009842	96	nucleotide at start position -1
0.009452	77	Triplet C(((
0.009243	42	Bulge at start position -1
0.009063	97	nucleotide at start position +1
0.008585	65	Triplet C(..
0.008039	51	Bulge at end position -1
0.007622	44	Bulge at start position -2
0.007532	99	nucleotide at end position -1
0.006332	3	Percentage of GC content
0.006258	49	Bulge at end position 0
0.004997	98	nucleotide at end position 0
0.004951	8	PresenceOfPerfect20MerBasePair
0.004951	9	Start of perfect 20 nt base pair in the miRNA
0.004827	71	U((.
0.003586	16	Percentage of G
0.003445	69	Triplet C((.
0.003346	22	Percentage of dinucleotides AU
0.002345	86	Triplet G..(
0.002264	82	Triplet G.(.
0.002256	54	Bulge at end position +3
0.002032	74	Triplet G.((
0.001912	26	Percentage of dinucleotides AG
0.001907	17	Percentage of C
0.001902	72	Triplet A.((
0.001868	57	Number of bulges
0.001827	70	Triplet G((.
0.001761	83	Triplet U.(.
0.001581	100	nucleotide at end position +1
0.001552	14	Percentage of dinucleotides A
0.00143	31	Percentage of dinucleotides UC
0.001339	28	Percentage of dinucleotides GG
0.001323	68	Triplet A((.
0.001287	87	Triplet U..(
0.001166	23	Percentage of dinucleotides UU
0.001134	48	Bulge at start position -4
0.001092	27	Percentage of dinucleotides UG
0.000989	88	Triplet A(.(
0.000912	15	Percentage of U
0.000905	25	Percentage of dinucleotides CU
0.00088	18	Percentage of dinucleotides AA
0.000861	46	Bulge at start position -3
0.000767	85	Triplet C..(
0.000765	81	Triplet C.(.
0.000754	33	Percentage of dinucleotides CC

0.000705	80	Triplet A.(.
0.000491	41	Bulge at start position 0
0.000415	50	Bulge at end position +1
0.000373	52	Bulge at end position +2
0	1	length
0	19	Percentage of dinucleotides UA
0	20	Percentage of dinucleotides GA
0	21	Percentage of dinucleotides CA
0	24	Percentage of dinucleotides GU
0	29	Percentage of dinucleotides CG
0	30	Percentage of dinucleotides AC
0	32	Percentage of dinucleotides GC
0	73	Triplet C.((
0	75	Triplet U.((
0	84	Triplet A..(
0	89	Triplet C(.(
0	90	Triplet G(.(
0	91	Triplet U(.(

Attribute ranking output for Plants

Rank scores	ID	Features
0.2183596	40	Average number of paired bases in a sliding widow of 3nt along the miRNA
0.2136904	2	Minimum free energy of the duplex
0.2134001	7	Bases pairs in the duplex of the miRNA and its complementarity region
0.2098282	39	Average number of paired bases in a sliding widow of 5nt along the miRNA
0.2029981	59	Length of the biggest bulges in percentage of the miRNA length
0.2001381	38	Average number of paired bases in a sliding widow of 7nt along the miRNA
0.1973837	58	Length of the biggest bulges in nucleotides
0.1552158	35	Distance of the start of miRNA from the start of the hairpin
0.1508973	34	Distance of the miRNA from the terminal loop of the hairpin
0.1241754	63	Triplet U...
0.1127512	60	Triplet A...
0.1121255	4	Maximum length without bulges
0.1101996	5	Maximum length without bulges in percentage of the miRNA length
0.1072775	37	Length of the miRNA which overlap in the hairpin loop
0.1022462	93	Percentage of GC base pairs in the duplex
0.0981264	13	Start of perfect 5 nt base pair in the miRNA
0.0897533	61	Triplet C...
0.0835688	94	Percentage of GU base pairs in the duplex
0.0826534	92	Percentage of AU base pairs in the duplex
0.0763566	36	miRNA included in loop
0.0709132	11	Start of perfect 10 nt base pair in the miRNA
0.0689285	62	Triplet G...
0.0583529	10	PresenceOfPerfect10MerBasePair
0.0581855	45	Bulge at start position 2
0.0581738	78	Triplet G(((
0.0527604	3	Percentage of GC content
0.0487617	6	Length without bulges from miRNA start
0.0485925	55	Bulge at end position -3
0.044935	95	nucleotide at start position 0
0.0396789	12	PresenceOfPerfect5MerBasePair
0.0373814	43	Bulge at position 1
0.0372496	57	Number of bulges
0.0366634	47	Bulge at start position 3
0.0305678	16	Percentage of G
0.0295102	53	Bulge at end position -2
0.0272025	77	Triplet C(((
0.0267036	64	Triplet A(..
0.0263878	67	Triplet U(..
0.0257003	9	Start of perfect 20 nt base pair in the miRNA
0.0246115	8	PresenceOfPerfect20MerBasePair
0.0224664	19	Percentage of dinucleotides UA
0.0220973	15	Percentage of U

0.0210338	18	Percentage of dinucleotides AA
0.020926	23	Percentage of dinucleotides UU
0.0199528	76	Triplet A(((
0.0195947	20	Percentage of dinucleotides GA
0.0181526	26	Percentage of dinucleotides AG
0.0175972	22	Percentage of dinucleotides AU
0.0161085	97	nucleotide at start position +1
0.015936	32	Percentage of dinucleotides GC
0.0155202	49	Bulge at end position 0
0.0148667	28	Percentage of dinucleotides GG
0.0146388	98	nucleotide at end position 0
0.0142824	44	Bulge at start position -2
0.0142462	87	Triplet U..(
0.0136328	51	Bulge at end position -1
0.0133243	65	Triplet C(..
0.0130869	100	nucleotide at end position +1
0.0120069	14	Percentage of dinucleotides A
0.011292	79	Triplet U(((
0.0101996	41	Bulge at start position 0
0.0092681	27	Percentage of dinucleotides UG
0.0082792	71	U((.
0.0080999	84	Triplet A..(
0.0071784	66	Triplet G(..
0.0064138	42	Bulge at start position -1
0.0063921	17	Percentage of C
0.0062835	33	Percentage of dinucleotides CC
0.0052242	21	Percentage of dinucleotides CA
0.0049397	24	Percentage of dinucleotides GU
0.0048082	85	Triplet C..(
0.0046388	68	Triplet A((.
0.0040023	29	Percentage of dinucleotides CG
0.0035412	86	Triplet G..(
0.0033861	69	Triplet C((.
0.0020496	99	nucleotide at end position -1
0.0020327	81	Triplet C.(.
0.001623	82	Triplet G.(.
0.0015835	73	Triplet C.((
0.0015628	75	Triplet U.((
0.001333	83	Triplet U.(.
0.0012765	52	Bulge at end position +2
0.0012623	89	Triplet C(.(
0.0011736	54	Bulge at end position +3
0.0010792	72	Triplet A.((
0.0008841	80	Triplet A.(.
0.000736	56	Bulge at end position +4
0.0006831	96	nucleotide at start position -1
0.0005589	46	Bulge at start position -3
0.0002641	50	Bulge at end position +1
0.0000113	48	Bulge at start position -4
0	1	length
0	25	Percentage of dinucleotides CU
0	30	Percentage of dinucleotides AC
0	31	Percentage of dinucleotides UC
0	70	Triplet G((.
0	74	Triplet G.((
0	88	Triplet A(.(
0	90	Triplet G(.(
0	91	Triplet U(.(

Supplementary Figure 1

Cumulative distribution of the distance between the true and predicted miRNAs starts and ends, i.e. the proportion of cases where the prediction is within *x* bases of the true start/end positions. We only show distances of up to 10nt, but in some rare cases errors are substantially larger (up to 250 nt). Results are presented for lineage-specific miRdup for mammals, arthropods, nematods, fish and plants.

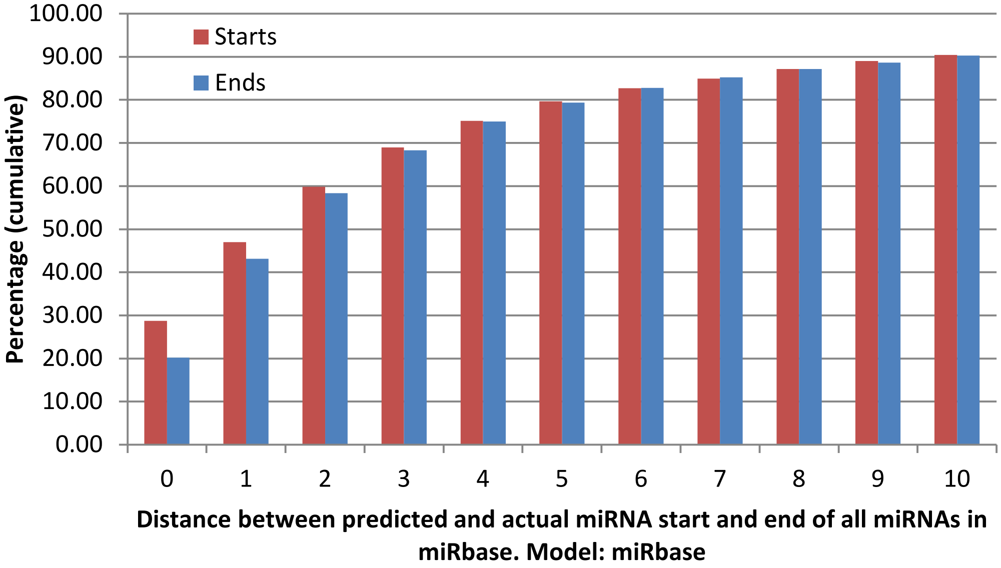

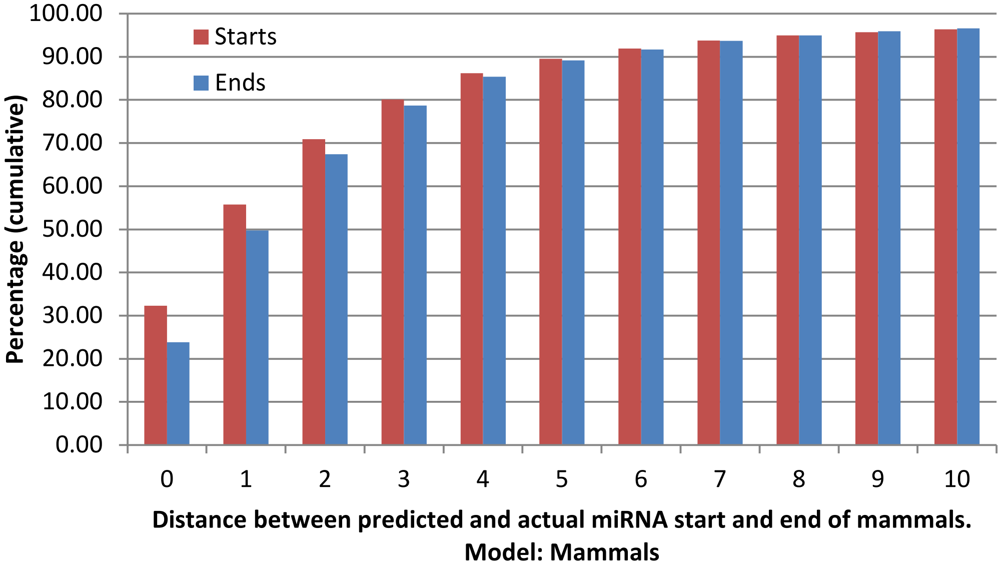

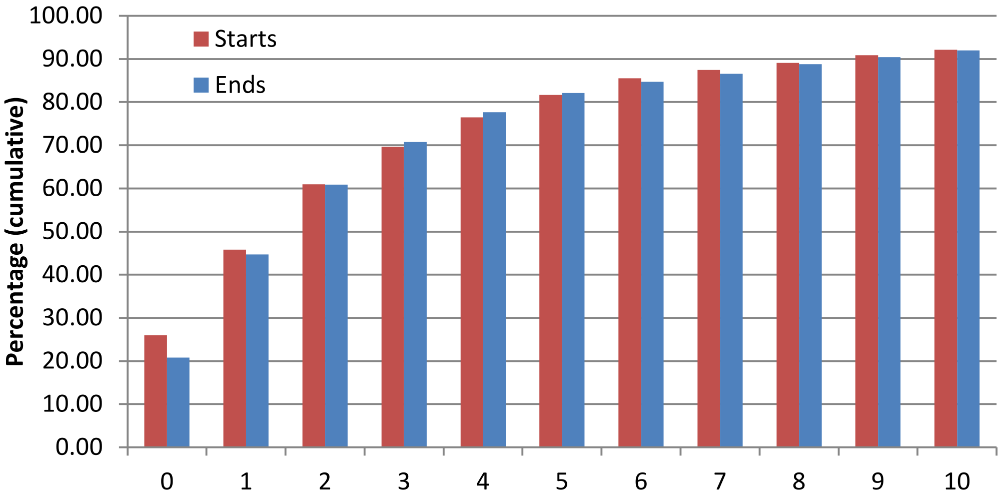

Distance between predicted and actual miRNA start and end of arthropods.
Model: Arthropods

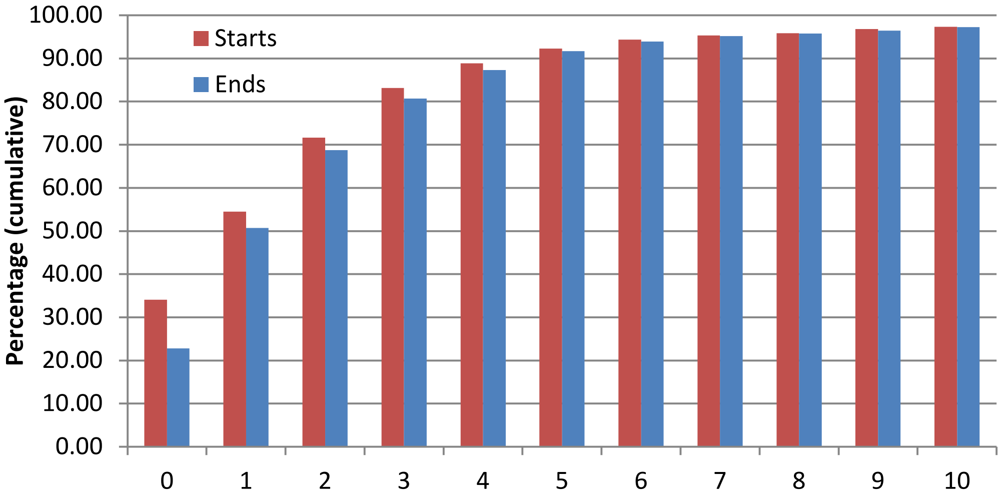

Distance between predicted and actual miRNA start and end of nematods.
Model: Nematods

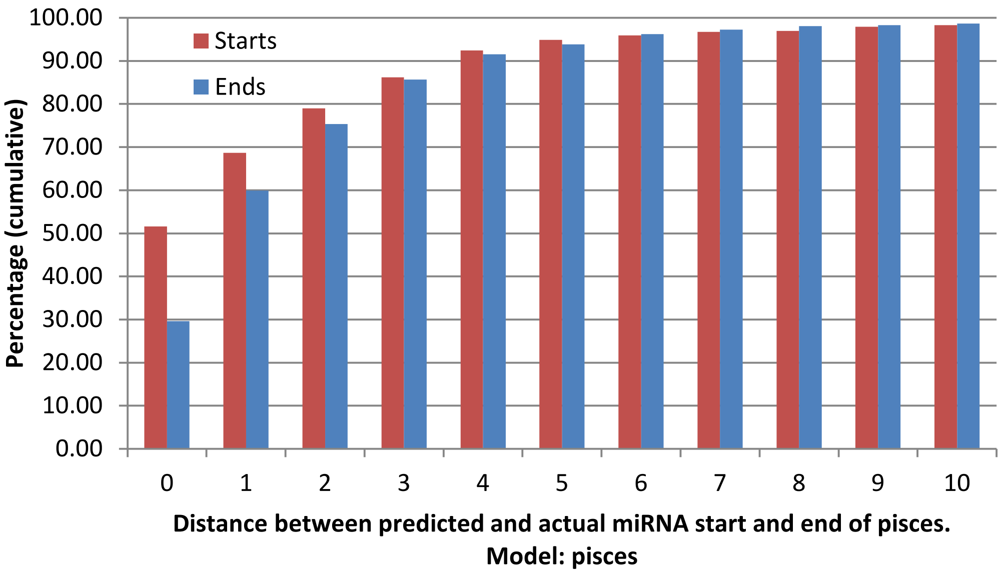

100.00
90.00
80.00
70.00
60.00
50.00
40.00
30.00
20.00
10.00
0.00
Starts
Ends
Percentage (cumulative)
0
1
2
3
4
5
6
7
8
9
10
Distance between predicted and actual miRNA start and end of pisces.
Model: pisces

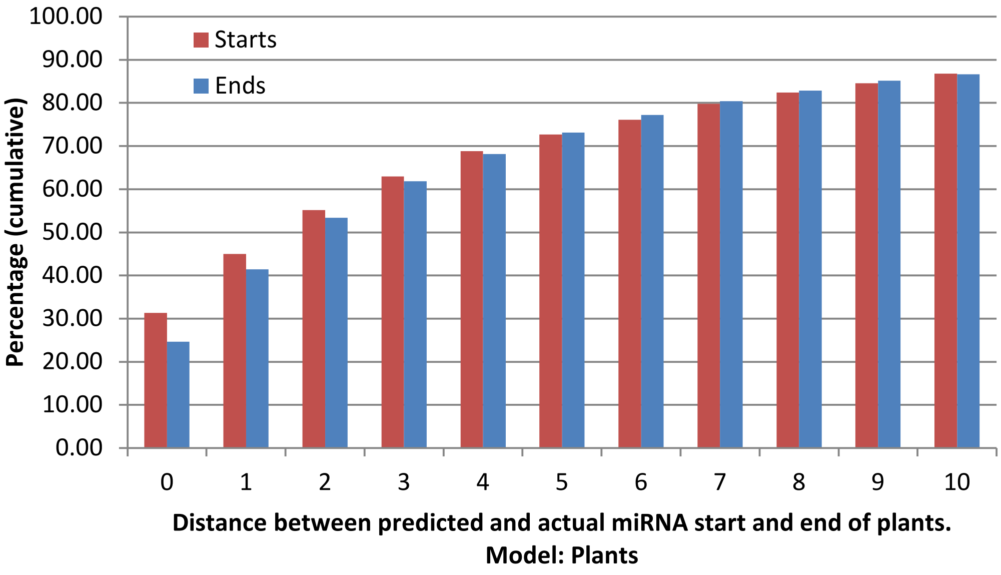

100.00
90.00
80.00
70.00
60.00
50.00
40.00
30.00
20.00
10.00
0.00
Starts
Ends
Percentage (cumulative)
0
1
2
3
4
5
6
7
8
9
10
Distance between predicted and actual miRNA start and end of plants.
Model: Plants
